# The Japanese Government’s “Good Sleep” Challenge: Sleep Guidelines for Health Promotion 2014

**DOI:** 10.2188/jea.JE20140217

**Published:** 2015-04-05

**Authors:** Hiroyuki Noda

**Affiliations:** 1 Cancer Control and Health Promotion Division, Health Service Bureau, Ministry of Health, Labour and Welfare, Tokyo, Japan; 2 Public Health, Department of Social and Environmental Medicine, Graduate School of Medicine, Osaka University, Suita, Osaka, Japan

Sleep is one of the most important components of lifestyles that are associated with risk of non-communicable diseases (NCDs). For example, it is well known that both short and long durations of sleep are associated with increased risk of cardiovascular disease.^1^ Some studies have shown that irregular sleep rhythms (eg, those resulting from shift work) is associated with increased risk of cardiovascular disease^2^ and cancer.^3^ Poor quality of sleep due to apnea and nocturnal intermittent hypoxia is also recognized as a risk factor for cardiovascular disease.^4^^,^^5^ Therefore, it is crucial to have “good sleep” for the prevention and control of NCDs; however, we have few guidelines for sleep, unlike other lifestyles, such as diet and physical activity. In fact, there is no description related to sleep in the global action plan for the prevention and control of NCDs for 2013–2020.^6^

At the end of March 2014, the Japanese Heath Service Bureau published “Sleep Guidelines for Health Promotion 2014”^7^ after thorough discussion by an expert committee for the revision of previous guidelines. First, the expert committee concluded that evidence exists, especially from epidemiological studies with large sample sizes of free-living populations over the last decade, that allows us to understand the effect of sleep on risk of NCDs and focusing on “good sleep” for health.

The new guideline has 12 messages on how to improve sleep for health, such as how sleep can reduce risk of NCDs as well as accidents. Although previous guidelines used the phrase “comfortable sleep”, the expert committee concluded that “comfortable sleep” was too subjective an expression and decided not to use this phrase in the new guideline.

The present guidelines have messages for three generations (ie, the young, middle-aged, and elderly). For the young generation, such as schoolchildren, the guidelines recommend not staying up late, as this may result in a short duration of sleep.^8^ The use of smartphones is also likely to delay the time of falling asleep and reduce sleep duration. We warned young persons and their parents, as well as health professionals, of these and other current major causes for inadequate sleep duration.

For the working middle-aged generation, the guidelines recommend that workers sleep as long as they can, because persons in the working generation are likely to receive inadequate sleep and justify it as being for work.^9^ To help workers improve their sleep, we also mention several tips for falling asleep and sleeping deeply.

Persons in the elderly generation were encouraged not to make an effort to extend sleep duration if they cannot sleep. Some elderly people get enough sleep but are obsessed with the idea that they could not sleep well, which leads to difficulty falling asleep.^10^ This message encourages them to have “good sleep” by reducing their obsession.

The Japanese Health Service Bureau published the new guideline to be used by health professionals in community workplaces and clinics, but it will also be made available to all residents through the government’s health promotion measures as well as commercial tools. We also requested that all users review their own sleep and use the guidance to improve specific individual sleep issues, because the adequate range of duration and suitable methods vary widely between individuals and generations.

The Government of Japan has started a new challenge for health promotion through “good sleep” for the prevention and control of NCDs. In this challenge, we encourage all residents to assess and improve their sleep though “Sleep Guidelines for Health Promotion 2014”.

## References

[1] Ikehara S, Iso H, Date C, Kikuchi S, Watanabe Y, Wada Y, ; JACC Study Group. Association of sleep duration with mortality from cardiovascular disease and other causes for Japanese men and women: the JACC study. Sleep. 2009;32:295–301. 19294949 10.1093/sleep/32.3.295 PMC2647783

[2] Vyas MV, Garg AX, Iansavichus AV, Costella J, Donner A, Laugsand LE, . Shift work and vascular events: systematic review and meta-analysis. BMJ. 2012;345:e4800. 10.1136/bmj.e4800 22835925 PMC3406223

[3] Wang F, Yeung KL, Chan WC, Kwok CC, Leung SL, Wu C, . A meta-analysis on dose-response relationship between night shift work and the risk of breast cancer. Ann Oncol. 2013;24:2724–32. 10.1093/annonc/mdt283 23975662 PMC7364178

[4] Dong JY, Zhang YH, Qin LQ. Obstructive sleep apnea and cardiovascular risk: meta-analysis of prospective cohort studies. Atherosclerosis. 2013;229:489–95. 10.1016/j.atherosclerosis.2013.04.026 23684511

[5] Tanigawa T. Obstructive sleep apnea: its prevention and screening may contribute to the prevention of hypertension, diabetes and cardiovascular diseases. EPMA J. 2011;2:83–9. 10.1007/s13167-011-0073-2 23199130 PMC3405377

[6] World Health Organization. Global action plan for the prevention and control of noncommunicable diseases 2013–2020. Geneva: WHO Document Production Services; 2013.

[7] Ministry of Health, Labour and Welfare, Health Service Bureau, ed. Sleep Guideline for Health Promotion 2014. Tokyo: Ministry of Health, Labour and Welfare, Health Service Bureau; 2014. Available: http://www.mhlw.go.jp/file/06-Seisakujouhou-10900000-Kenkoukyoku/0000047221.pdf.

[8] Fukuda K, Ishihara K. Age-related changes of sleeping pattern during adolescence. Psychiatry Clin Neurosci. 2001;55:231–2. 10.1046/j.1440-1819.2001.00837.x 11422853

[9] Ministry of Health, Labour and Welfare. Survey on Health and Welfare Trend Heisei 12. Tokyo: Ministry of Health, Labour and Welfare; 2002.

[10] Yoshiike T, Kuriyama K, Honma M, Ikeda H, Kim Y. Neuroticism relates to daytime wakefulness and sleep devaluation via high neurophysiological efficiency in the bilateral prefrontal cortex: a preliminary study. Psychophysiology. 2014;51:396–406. 10.1111/psyp.12180 24660887

